# A Multitarget Therapeutic Peptide Derived From Cytokine Receptors Based on in Silico Analysis Alleviates Cytokine-Stimulated Inflammation

**DOI:** 10.3389/fphar.2022.853818

**Published:** 2022-03-10

**Authors:** Chun-Chun Chang, Shih-Yi Peng, Hao-Hsiang Tsao, Hsin-Ting Huang, Xing-Yan Lai, Hao-Jen Hsu, Shinn-Jong Jiang

**Affiliations:** ^1^ Department of Laboratory Medicine, Hualien Tzu Chi Hospital, Hualien, Taiwan; ^2^ Department of Laboratory Medicine and Biotechnology, College of Medicine, Tzu Chi University, Hualien, Taiwan; ^3^ Department of Biochemistry, School of Medicine, Tzu Chi University, Hualien, Taiwan; ^4^ Department of Life Sciences, College of Medicine, Tzu Chi University, Hualien, Taiwan

**Keywords:** sepsis, cytokine, inflammation, peptide drug, reactive oxygen species, tumor necrosis factors (TNF)-α

## Abstract

Septicemia is a severe inflammatory response caused by the invasion of foreign pathogens. Severe sepsis-induced shock and multiple organ failure are the two main causes of patient death. The overexpression of many proinflammatory cytokines, such as TNF-α, IL-1β, and IL-6, is closely related to severe sepsis. Although the treatment of sepsis has been subject to many major breakthroughs of late, the treatment of patients with septic shock is still accompanied by a high mortality rate. In our previous research, we used computer simulations to design the multifunctional peptide KCF18 that can bind to TNF-α, IL-1β, and IL-6 based on the binding regions of receptors and proinflammatory cytokines. In this study, proinflammatory cytokines were used to stimulate human monocytes to trigger an inflammatory response, and the anti-inflammatory ability of the multifunctional KCF18 peptide was further investigated. Cell experiments demonstrated that KCF18 significantly reduced the binding of proinflammatory cytokines to their cognate receptors and inhibited the mRNA and protein expressions of TNF-α, IL-1β, and IL-6. It could also reduce the expression of reactive oxygen species induced by cytokines in human monocytes. KCF18 could effectively decrease the p65 nucleus translocation induced by cytokines, and a mice endotoxemia experiment demonstrated that KCF18 could reduce the expression of IL-6 and the increase of white blood cells in the blood stimulated by lipopolysaccharides. According to our study of tissue sections, KCF18 alleviated liver inflammation. By reducing the release of cytokines in plasma and directly affecting vascular cells, KCF18 is believed to significantly reduce the risk of vascular inflammation.

## Introduction

Cytokines are mostly 8–25 kDa water-soluble proteins and glycoproteins that are involved in cell signaling. For their main functions, cytokines promote cell proliferation, activation, and differentiation and participate in inflammation, immune response regulation, and tissue repair (Cannon, 2000). Cytokines include chemokines, interferons (IFNs), interleukins (ILs), and tumor necrosis factors (TNFs). When the human body is infected by pathogens, it causes macrophages to secrete numerous proinflammatory cytokines, such as IL-1, IL-6, IL-8, and TNF-α. These proinflammatory cytokines facilitate the activation of white blood cells; they prompt the accumulation of white blood cells, enhance the activation of endothelial cells, increase the permeability of blood vessels, produce inflammatory responses, remove foreign pathogens, and induce the generation of acquired immune responses. Therefore, constant cytokines are crucial for maintaining body health. However, when cytokines are overexposed, they may cause cell damage and further injury to organs. This phenomenon is called a cytokine storm, which eventually leads to the occurrence of diseases (Kilroy et al., 2007; Sun et al., 2012; D’elia et al., 2013) such as sepsis, cytokine release syndrome, and systemic inflammatory response syndrome.

Reactive oxygen species (ROS) are highly biologically active substances produced during the process of oxidative metabolism in the human body (Devasagayam et al., 2004; Pacher et al., 2007) and are exemplified by molecules such as superoxide radicals (O2^−•^), hydroxyl radicals (OH^•^), and hydrogen peroxide (H_2_O_2_). An appropriate amount of ROS is involved in cellular signal transduction and regulation of cell growth (Radi, 2013). In addition, white blood cells and macrophages can produce O2^−•^, H_2_O_2_, and other ROS to eliminate foreign pathogens (Prolo et al., 2014). However, excessive ROS will destroy the integrity of the antioxidant defense system in the body and cause oxidative stress and oxidative damage in the organism (Radi, 2013; Prolo et al., 2014); for example, 1) ROS may attack the polyunsaturated fatty acids on the cell membrane of the body, causing lipid peroxidation and then destroying the integrity of cell membranes. 2) ROS may change the protein structure or denature proteins, leading to a loss of enzyme activity that catalyzes metabolic reactions. 3) They may damage the base structure of DNA molecules and further cause gene mutations or toxicity. 4) ROS may stimulate white blood cells and macrophages to release cytokines and cause inflammation. These reactions can cause cell damage, destruction, and even cell death. The destruction and accumulation of these ROS are key reasons for the occurrence of aging-related phenomena and diseases, such as degenerative diseases, cancer, cardiovascular disease, diabetes, and autoimmune disease, as described in many studies (Muller et al., 2007; Sinha et al., 2014). Although oxidative stress is considered to be a phenomenon that accompanies inflammation, increasing evidence has shown that oxidative stress can be an early stage in the development of inflammation-related diseases (West et al., 2011; Kvietys and Granger, 2012; Craige et al., 2015). The clearance of ROS plays a vital role in the prevention and treatment of inflammation-related diseases. NF-κB is a significant transcription factor in the signal transmission pathway and is closely related to inflammation, apoptosis, and tumorigenesis (Yamamoto and Gaynor, 2001; Lawrence, 2009). NF-κB is a dimer composed of various subunits, including p50, p52, p65 (RelA), RelB, and c-Rel. It regulates the expression of cytokines, chemoattractant factors, and adhesion molecules. NF-κB is mainly a heterodimer formed by p50 and p65, and it usually combines with IκB to form an inactive form in cytoplasm. When NF-κB is activated by cytokines (such as IL-1β or TNF-α), proinflammatory substances (such as ROS), or bacterial products (such as lipopolysaccharides [LPS]), IκB will be phosphorylated and separated from NF-κB, which enables NF-κB to enter the cell nucleus; moreover, phosphorylated p65 can bind to DNA for transcriptional regulation, which consists of 1) promoting the expression of proinflammatory cytokines and chemoattractant factors such as TNF-α, IL-1, and IL-6, which activate immune cells. Additionally, the transcriptional regulation 2) may entail the induction of adhesion molecules, such as ICAM, VCAM, E-selectin, and P-selectin, to promote the migration of immune cells and 3) increasing the expression of COX-2 and iNOS to exacerbate inflammatory responses. The increase in the amount of NF-κB is closely related to the severity of the inflammatory reaction (Lawrence, 2009).

In recent years, the possibility of developing peptides into therapeutic drugs has gradually come to be valued by many scientists (Otvos and Wade, 2014). Numerous pharmaceutical and biotech companies are engaged in developing peptide drugs, mainly because peptide drugs have comparative advantages in affinity and cell penetration—superior to natural proteins (Otvos and Wade, 2014; Fosgerau and Hoffmann, 2015)—which can trigger or inhibit the occurrence of signal transmission, overcome the limitations of existing drugs, and improve permeability for entering tissues. In addition, peptide drugs have predictable metabolic pathways, safety, and tolerability; hence, researchers can determine whether peptide drugs will cause damage to other tissues and organs. By contrast, the disadvantage of peptide drugs is their short half-life in the blood, which may render them unable to achieve the purpose of treatment (Komin et al., 2017). Therefore, the improvement and design of peptide drugs are of critical importance.

At present, the treatment of sepsis is mainly based on antibiotic treatment, cardiovascular and pulmonary support therapy, and other treatment methods intended to maintain the normal physiological function of a patient’s body. No effective treatment method can reduce the cytokine storm induced by sepsis. KCF18 is a multifunctional peptide, the design of which is mainly based on the receptors of TNF-α, IL-1β, and IL-6. In previous computer simulations and surface plasmon resonance experiments, KCF18 has been proven to bind to TNF-α, IL-1β, and IL-6 simultaneously and to effectively reduce the adhesion of monocytes induced by LPS (Jiang et al., 2019). The adhesion of monocytes is closely related to the expression of proinflammatory cytokines in blood vessels (Chang et al., 2012). The KCF18 peptide may be able to bind to these proinflammatory cytokines and reduce the stimulation of cytokines to alleviate the severity of sepsis. In this study, we investigated how the KCF18 peptide modulates the functional changes of macrophages under the inflammatory response induced by cytokines and decreases the severity of sepsis in an endotoxemia mouse model.

## Materials and Methods

### Cell Culture

The human monocytic cell line THP-1 was cultivated in RPMI-1640 medium containing 10% fetal bovine serum (FBS). The human microvascular endothelial cells HMEC-1 (ATCC, No CRL-10636) were maintained in MCDB131 medium containing endothelial cell growth supplement (Millipore, Billerica, MA, United States) and 15% FBS, as previously described (Dinarello, 2000).

### Cell Viability Assays

THP-1 (2 × 10^4^ cells/well) cells were grown in 96-well plates overnight. In the beginning, a medium containing KCF18 at various concentrations was added. After 24 and 48 h incubation periods, the viability of cells was evaluated using the WST-1 assay (Roche, Indianapolis, IN, United States), according to the manufacturer’s instructions.

### Cytokine Assays

THP-1 cells were pretreated with PMA (40 nM) for 24 h to differentiate into macrophages. After 24 h, the cells were washed three times with 1×HBSS. PMA-pretreated cells were cultivated with cytokines at a concentration of 20 ng/ml or cytokines pretreated with KCF18 for 1 h. Cultures were incubated for 24 h at 37°C. After stimulation, conditioned medium was collected, and TNF-α, IL-6, and IL-1β were detected using commercial ELISA kits.

### Measurement of ROS Production

Intracellular ROS production was assayed using 2,7-diclorofluorescein diacetate (H2DCFDA; Invitrogen). Cytokines were pretreated with KCF18 for 1 h and then incubated with THP-1 cells for 20 min. THP-1 cells were then cultivated with H2DCFDA (10 μM) for 5 min at 37°C. Images were obtained using a fluorescence microscope (IX-71, Olympus). Fluorescence intensity was obtained using ImageJ software, averaged, and normalized to the control value.

### Oxidized Low-Density Lipoprotein Uptake Assay

Low-density lipoprotein (LDL) was added to 5 µM CuSO_4_, and the mixture was incubated in an incubator (37°C, 5% CO_2_) for 24 h to oxidize the LDL. Subsequently, 250 μM EDTA was added to stop the oxidation reaction, and the mixture was stored in a 4°C refrigerator. THP-1 cells (3 × 10^5^ cells) were added into a 12-well plate (1 ml/well) and cocultured with 50 μg/ml oxLDL in serum-free medium for 24 h to promote oxLDL phagocytosis by macrophages. After 24 h, the cells were washed with PBS, and 4% paraformaldehyde (1 ml/well) was added to fix the cells at room temperature for 1 h. After 1 h, the paraformaldehyde was removed, and the cells were washed with ddH_2_O. Sixty percent isopropanol (500 µL/well) was added at room temperature for 5 min. Subsequently, the isopropanol was removed, Oil Red O dye (500 µL/well) was added, and the cell mixture was incubated at room temperature for 2 h. After 2 h, the cells were washed with ddH_2_O, and the image thereof was taken with a Zeiss Axio Vert A1 fluorescent microscope. Ethyl alcohol (99.5%, 500 µL/well) was added to the cells to extract the Oil Red O. The Thermo Scientific Multiskan Spectrum was used to detect absorbance at 540 nm.

### Transient Transfection and Luciferase Assays

Endothelial cells at 80% confluence were transiently transfected with plasmids using Lipofectamine (Invitrogen), according to the manufacturer’s protocol. Briefly, 0.5 μg of pGL3-4 kB-Luc or 0.1 μg of pCMV-β-gal was mixed with the Lipofectamine reagent; the mixture was then added to cells for 6 h. Nontransfected reagents were washed, and culture medium was added to cells. After 18 h, cytokines were pretreated with KCF18 for 1 h and incubated with cells for 6 h. Lysis buffer was added to the cells to lyse them, and luciferase and β-galactosidase activities were assessed using a luciferase assay kit (Promega, Madison, WI, United States) according to the manufacturer’s instructions. Luciferase activity was normalized with respect to β-galactosidase activity and expressed as a percentage of control activity.

### RNA Isolation and Quantitative Polymerase Chain Reaction Assays

The HMEC-1 and THP-1 cells were grown to confluence in 6 cm^2^ culture plates and then treated with cytokines at a concentration of 20 ng/ml or cytokines pretreated with KCF18 for 1 h. Cultures were incubated for 4 h at 37°C. Total RNA was isolated using Trizol Reagent (Invitrogen), according to the manufacturer’s suggested protocol. An aliquot (5 μg) of purified RNA was reverse transcribed into first-strand complementary DNA (cDNA) by using a 2,720 Thermal Cycler (Applied Biosystems, Grand Island, NY, United States), 200 U/μL M-MLV reverse transcriptase (Invitrogen), and 0.5 mg/μL oligo (dT)-adapter primers (Invitrogen) in a 20-μL reaction mixture. The quantitative polymerase chain reaction (qPCR) assays for TNF-α, IL-1β, IL-6, and glyceraldehyde 3-phosphate dehydrogenase (GAPDH) were performed with a Roche LightCycler 480 System (Roche, Indiana CA, United States). The oligonucleotide primers used were specific for TNF-α (5ʹ-AGG GAC CTC TCT CTA ATC AG-3ʹ and 5ʹ-TGG GAG TAG ATG AGG TAC AG-3ʹ), IL-1β (5ʹ-AAA CAG ATG AAG TGC TCC TTC-3ʹ and 5ʹ-TGG AGA ACA CTT GTT GCT-3ʹ), IL-6 (5ʹ-GCC GCC CCA CAC AGA CA-3ʹ and 5ʹ-CCG TCG AGG ATG TAC CGA AT-3ʹ), and GAPDH (5ʹ-ACG GAT TTG GTC GTA TTG GG-3ʹ and 5ʹ-TGA TTT TGG AGG GAT CTC GC-3ʹ). The oligonucleotide primers used for mice TNF-α (5ʹ-GCT CCC TCT CAT CAG TTC TAT-3ʹ and 5ʹ-TTT GCT ACG ADC TGG GCT A-3ʹ), IL-1β (5ʹ-CAA CCA ACA AGT GTA TTC TCC AT-3ʹ and 5ʹ-GTG TGC CGT CTT TCA TTA-3ʹ), IL-6 (5ʹ-GCT ACC AAA CTG GAT ATA ATC AGG-3ʹ and 5ʹ-CCA GGT AGC TAT GGT ACT CCA GAA-3ʹ), MCP-1 (5ʹ-CCC CAG TCA CCT GCT GTT AT-3ʹ and 5ʹ-CCA CAA TGG TCT TGA AGA TCA C-3ʹ), and GAPDH (5ʹ-TTC ACC ATG GAG AAG-3ʹ and 5ʹ-GGC ATG GAC TGT GGT CAT GA-3ʹ). Thermal cycling conditions involved an initial denaturation step at 95 °C followed by 35 amplification cycles (15 s at 95°C and 20 s at 60°C) and subsequent melt curve analysis (72–98°C). Quantitation of gene expression was conducted, with expression assessed relative to GAPDH expression levels.

### Animals and Endotoxemia Model

The animal experiments used 8-to-10-week-old male BABL/c mice as an animal model. These mice were purchased from the National Laboratory Animal Breeding and Research Center, Taipei. The mice were housed in a temperature-controlled, light-cycled facility, and this study was conducted in strict accordance with the recommendations of the Guide for the Care and Use of Laboratory Animals of the National Institutes of Health. The animal experiments performed by Dr. Shih-Yi Peng were permitted by the Tzu Chi University Institutional Animal Care and Use Committee (Permit Number: 101070). For *in vivo* experiments, mice received a single dose (10 mg/kg) of LPS (*Escherichia coli* 0,111:B4, SIGMA) administered *i. v.* After 4 h, the mice were injected with normal saline or 5 mg/kg KCF18 peptide. At 18 h after LPS injection, the mice were euthanized through exposure to CO_2_.

### Determination of Serum IL-6 in Mice

Blood was collected, and serum was separated through centrifugation (4°C, 3,000 rpm, 10 min). The levels of serum IL-6 were determined using the sandwich ELISA method according to the instructions provided with the reagent kits. The values are expressed as pg/mL of sera based on the appropriate standard curve.

### Histopathological Observation of Liver Tissues

The liver tissues of mice were fixed in 10% neutral phosphate buffered formalin solution, and they were then dehydrated and embedded in paraffin to make conventional paraffin sections. The sections were cut to be 4-μm thick and stained with hematoxylin and eosin (H&E). The histopathological changes of liver tissues were observed under an optical microscope.

### Statistical Analysis

The results are expressed as the means ± SD from at least three independent experiments. Differences between groups were assessed using a one-way analysis of variance. *p* < 0.05 was considered statistically significant.

## Results

### Molecular Surface Distribution of Peptides KCF18 and Mutant KCF18

**FIGURE 1:**
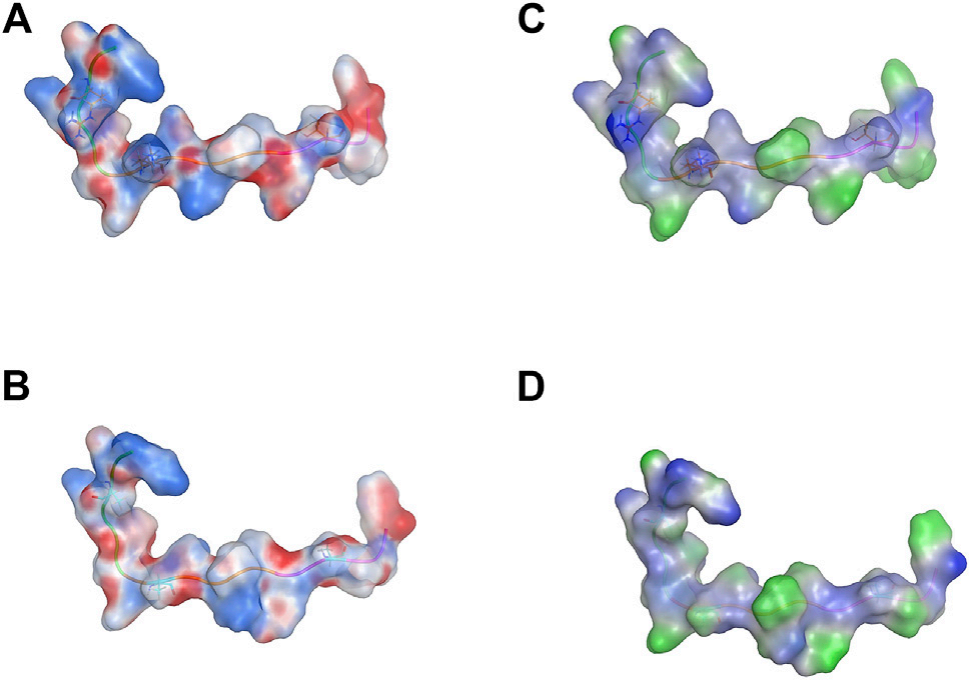
Molecular surface distribution of designed peptides KCF18 and mutant KCF18. Molecular surface charge distribution of **(A)** KCF18 and **(B)** mutant KCF18, which was calculated using the Poisson–Boltzmann equation in MOE 2020.09 (Molecular Operating Environment, http://www.chemcomp.com). Blue corresponds to positive, and red corresponds to negative electrostatic potential. KCF18 is more positively charged than mutant KCF18. Lipophilicity distribution of **(C)** KCF18 and **(D)** mutant KCF18, where blue represents hydrophilic residues, and green represents hydrophobic residues.

The designed combinational peptide KCF18 is truncated from the binding regions of cytokine receptors to cytokines (TNF-α, IL-1β, and IL-6), as shown in our previous study (Jiang et al., 2019). The molecular surface charge distribution of KCF18 indicated amphiphilic properties, with one side more positively charged and the other more negatively charged (Figure 1A). The lipophilicity of KCF18 was also more hydrophilic on one side and more hydrophobic on the other side (Figure 1C). However, the mutant peptide KCF18 (mKCF18) with three residues mutated to alanine (R3A, K8A, and T15A) exhibited fewer positively charged and more hydrophobic regions in the molecular surface distributions of charge and lipophilicity (Figures 1B,D). The mutant KCF18 exhibited less binding affinity to these cytokines than wild KCF18 and the specificity of KCF18 binding to these three cytokines was further validated in the following cellular experiments.

### Anti-Inflammatory Effect of KCF18 on TNF-α-, IL-1β-, and IL-6-Induced Proinflammatory Cytokine Transcription and Expression

**FIGURE 2:**
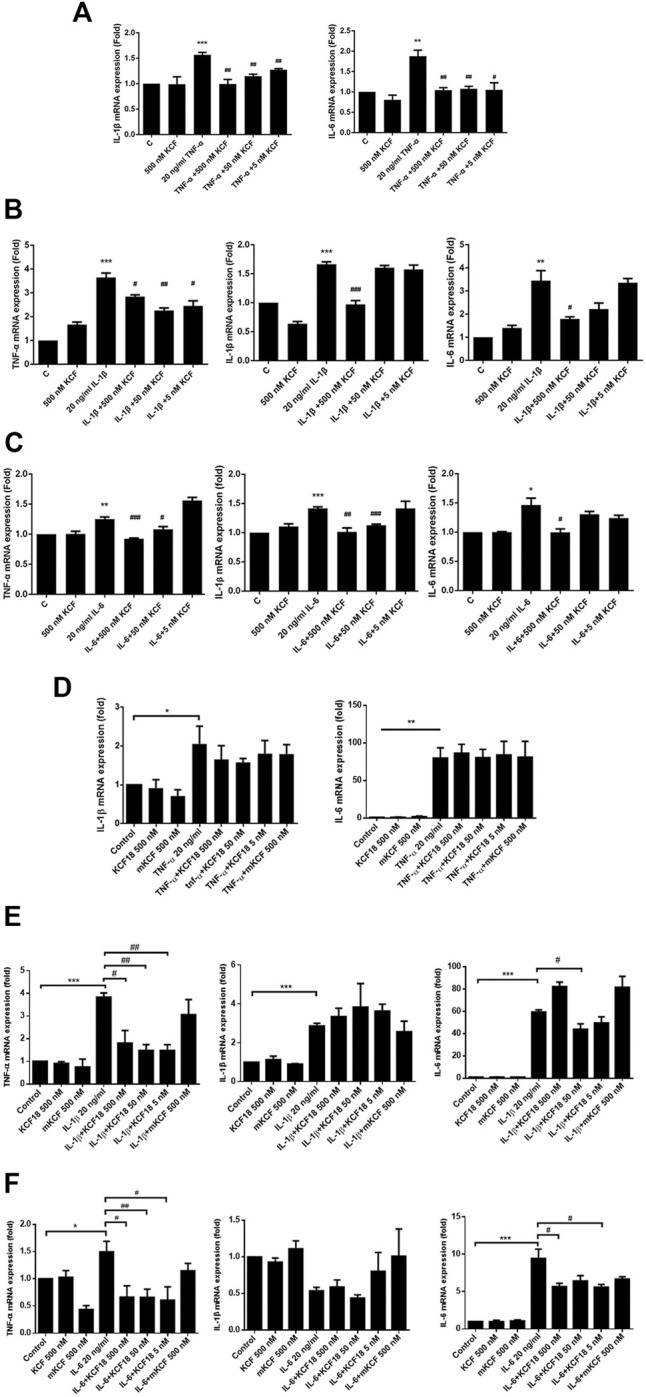
KCF18 downregulates cytokine-induced mRNA transcripts of TNF-α, IL-1β, and IL-6 in cells. PMA-pretreated THP-1 cells **(A–C)** or HMEC-1 cells **(D–F)** were cultivated with either cytokines at a concentration of 20 ng/ml or cytokines pretreated with KCF18 for 1 h. Four hours later, total RNA was isolated, and cytokine mRNA levels were determined using qPCR assays. GAPDH cDNA was used as an internal control. Values are the mean ± SD of mRNA levels relative to those for GAPDH from three independent experiments. *p < 0.05, **p < 0.01, ***p < 0.001 versus control, and #*p* < 0.05, ##*p* < 0.01, ###*p* < 0.001 versus cells stimulated with cytokines in the presence of KCF18.

**FIGURE 3:**
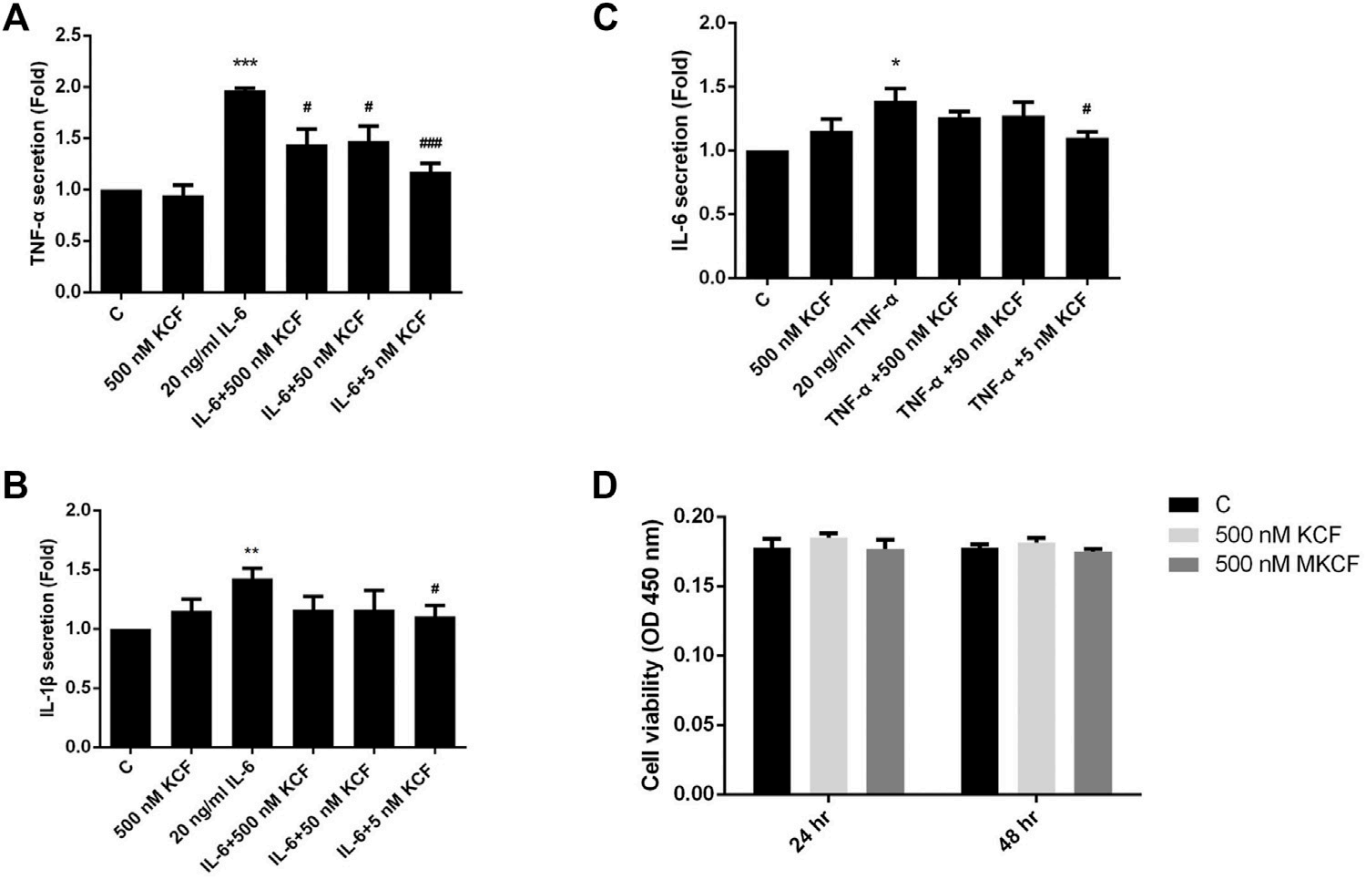
KCF18 reduces cytokine-induced secretions of TNF-α, IL-1β, and IL-6 in cells. PMA-pretreated THP-1 cells were incubated with either cytokines or cytokines pretreated with KCF18 for 1 h. Conditioned medium was collected 24 h later, and TNF-α **(A)**, IL-1β **(B)**, and IL-6 **(C)** secretions were detected. **(D)** THP-1 cells in a 96-well microplate were treated with various concentrations of KCF18. After a 24 and 48 h incubation, cell viability was evaluated using the colorimetric WST-1 assay. Values are the mean ± SD from three independent experiments.

To investigate whether KCF18 affects the expression of cytokines by endothelial cells and macrophages, proinflammatory cytokines (TNF-α, IL-1β, or IL-6) were pretreated with KCF18 for 1 h to bind the proinflammatory cytokines. After 1 h, the coculture medium was added to the cells, and mRNA was extracted after 4 h qPCR assays were used to quantify the effects of KCF18 at various concentrations on the expression of TNF-α, IL-1β, and IL-6 mRNA. Compared with the control cells, TNF-α, IL-1β, and IL-6 mRNA levels were significantly increased when macrophages (Figures 2A–C) and endothelial cells (Figures 2D–F) were cultured with TNF-α (Figures 2A,D), IL-1β (Figure 2Β and E), and IL-6 (Figures 2C,F). We also performed the experiments of TNF-α mRNA expression in cells stimulated with TNF-α (Supplementary Figure S1) and the results were similar with our previous publication (Shih et al., 2021). However, KCF18 significantly decreased cytokine-mediated expression of TNF-α, IL-1β, and IL-6 mRNA. In addition, no significant difference was observed in the mRNA expression levels between the KCF18-treated and control cells (Figure 2). The conditioned medium from 24 h was also collected to detect the secretion of TNF-α, IL-1β, and IL-6 in macrophages by using ELISA. As depicted in Figure 3, when IL-6 was added, the TNF-α (Figure 3A) and IL-1β (Figure 3B) secretions of macrophages increased significantly. TNF-α also induced IL-6 expression in macrophages (Figure 3C). By incubating with various concentrations of KCF18, the expression level of IL-6 was reduced, exhibiting a downward trend (Figure 3C). In the results for IL-6 stimulation, the secretion of TNF-α was upregulated nearly 200% by macrophages; however, 500, 50, and 5 nM KCF18 decreased the expression levels of TNF-α by 27, 25, and 40%, respectively (Figure 3A; *p* < 0.05 and 0.005). KCF18 also had the similar effects on IL-6 induced IL-1β expression (Figure 3B) and TNF-α induced IL-6 secretion (Figure 3C). The results indicated that KCF18 can bind to TNF-α, IL-1β, and IL-6, thereby reducing the activating effects of these cytokines on macrophages. To detect whether KCF18 is cytotoxic to monocytes, 500 nM KCF18 and mKCF peptides were added to THP-1 cells for 24 and 48 h, and cell viability was measured using WST-1 reagent. As depicted in Figure 3D, no significant difference between the control, KCF18, and mKCF groups was evident, confirming that 500 nM KCF18 and mKCF did not cause cytotoxicity to both cell lines. Hence, causing decreased cell viability is not the mechanism by which KCF18 acts on TNF-α, Il-1β, and IL-6 to reduce their induced mRNA transcript and protein expression.

### Inhibitory Effect of KCF18 on the Production of Cytokine-Induced ROS in Macrophages

**FIGURE 4:**
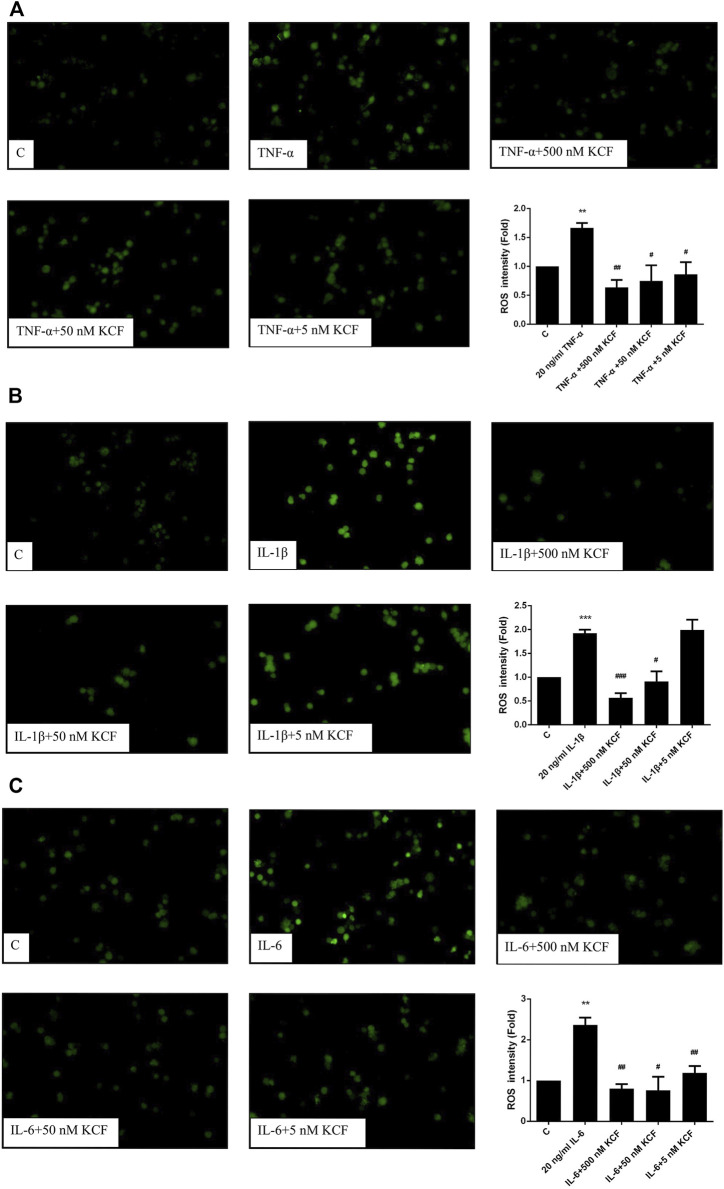
KCF18 reduces the production of cytokine-induced ROS in macrophages. ROS production induced by TNF-α **(A)**, IL-1β **(B)**, and IL-6 **(C)** was detected as described in the Materials and Methods section. Values are the mean ± SD of fluorescent intensities versus control from three independent experiments. **p < 0.01 versus control and #*p* < 0.05, ##*p* < 0.01 versus cells stimulated with cytokines in the presence of KCF18.

Many studies have shown that overexpression of ROS is closely related to inflammation (Kvietys and Granger, 2012). Therefore, we further explored whether KCF18 can ameliorate the oxidative stress induced by TNF-α, IL-1β, and IL-6 by detecting the level of intracellular ROS production. As predicted, TNF-α, IL-1β, and IL-6 treatment induced the fluorescence intensity of ROS in the cells, increasing the intensity by 1.66-, 1.92-, and 2.37-fold, respectively (Figures 4A–C). However, 500, 50, and 5 nM KCF18 pretreatment decreased the TNF-α-induced ROS expression in a dose-dependent manner to 0.64-, 0.78-, and 0.87-fold, respectively (Figure 4A; *p* < 0.01 and 0.05). KCF18 pretreatment reduced IL-1β-induced ROS expression by 0.57-fold and 0.91-fold, respectively (Figure 4B; 500 and 50 nM, *p* < 0.005 and 0.05). KCF18 pretreatment also reduced IL-6-induced ROS production by 0.81-, 0.76-, and 1.2-fold (Figure 4C; 500, 50, and 5 M, *p* < 0.01 and 0.05). The preceding results revealed that KCF18 can inhibit TNF-α-, IL-1β-, and IL-6-induced ROS production by macrophages.

### Effect of KCF18 on Cytokine-Induced Phagocytosis of oxLDL by Macrophages

**FIGURE 5:**
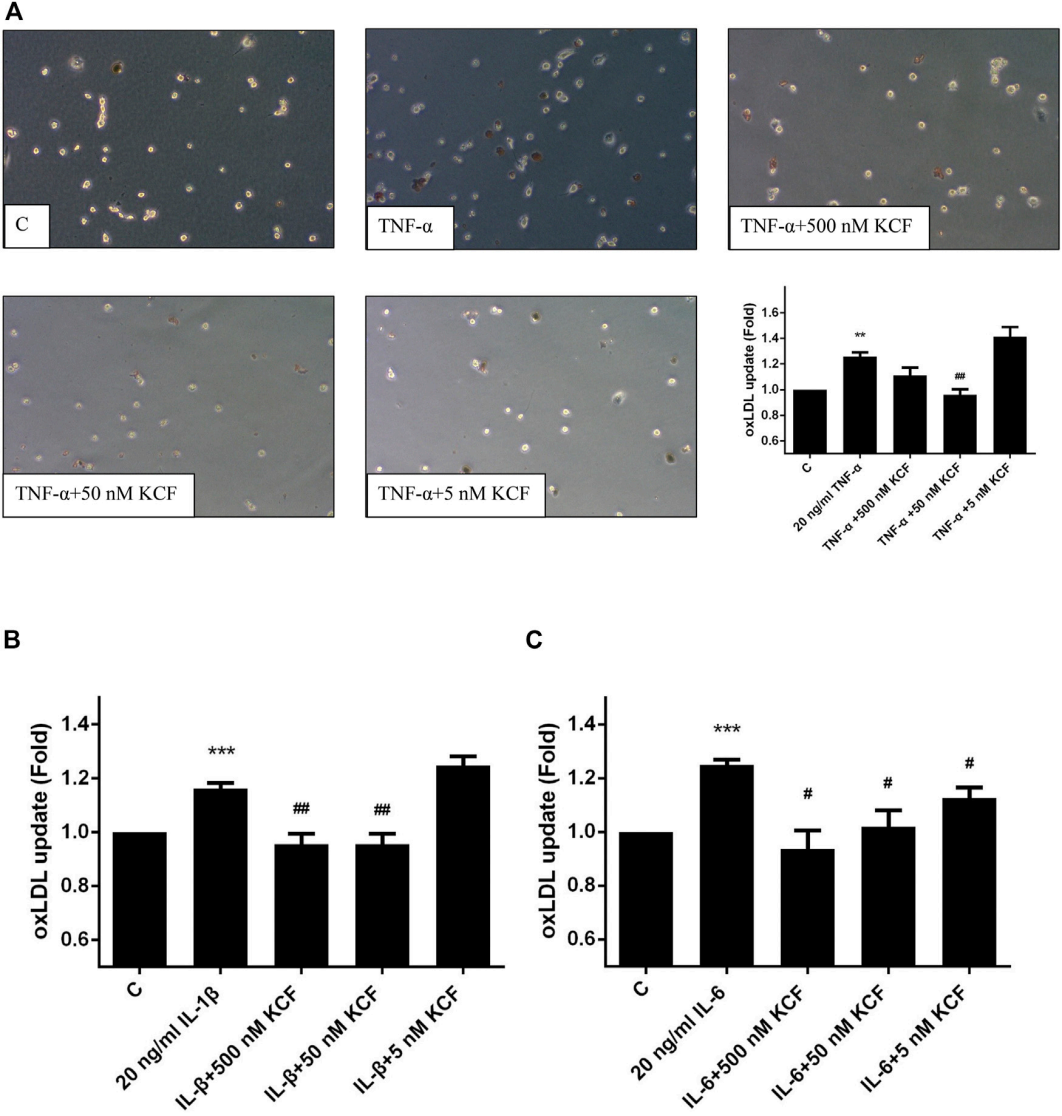
KCF18 decreases cytokine-induced phagocytosis of oxLDL by macrophages. oxLDL engulfment induced by TNF-α **(A)**, IL-1β **(B)**, and IL-6 **(C)** was determined. Values are the mean ± SD of fluorescent intensities versus control from three independent experiments. **p < 0.01, ***p < 0.001 versus control and #*p* < 0.05, ##*p* < 0.01 versus cells stimulated with cytokines in the presence of KCF18.

The oxLDL that accumulates on the inner wall of blood vessels can cause damage to endothelial cells and induce secretion of cytokines, attract monocytes to the inner wall of blood vessels, and differentiate into macrophages to phagocytose oxLDL. When macrophages converge into foam cells, which accumulate on the inner wall of blood vessels to form fibrotic plaque, it increases the risk of atherosclerosis (Bobryshev et al., 2016). We further validated whether KCF18 modulates the ability of macrophages to engulf oxLDL under the inflammation induced by cytokines. Figure 5A demonstrates that the phagocytosis of oxLDL by macrophages was increased by 1.26-fold after TNF-α stimulation. By contrast, 500 and 50 nM KCF18 decreased the oxLDL engulfment by 1.11-fold and 0.96-fold, respectively (*p* < 0.01). Furthermore, KCF18 reduced the phagocytosis of oxLDL by macrophages induced by IL-1β and IL-6 (Figures 5B,C). The amount of oxLDL phagocytosed by macrophages increased by 1.16-fold when IL-1β was added, whereas 500 and 50 nM KCF18 pretreatment reduced oxLDL phagocytosis to 0.96-fold (*p* < 0.01). IL-6 stimulated macrophages to phagocytose oxLDL 1.25-fold, whereas various concentrations of KCF18 (500, 50, and 5 nM) decreased oxLDL engulfment by 0.94, 1.02, and 1.12 times, respectively (*p* < 0.05). The results corroborated that KCF18 could alleviate cytokine-induced oxLDL phagocytosis by macrophages.

### Inhibitory Effect of KCF18 on the Nuclear Translocation of p65 Stimulated by Cytokines

**FIGURE 6:**
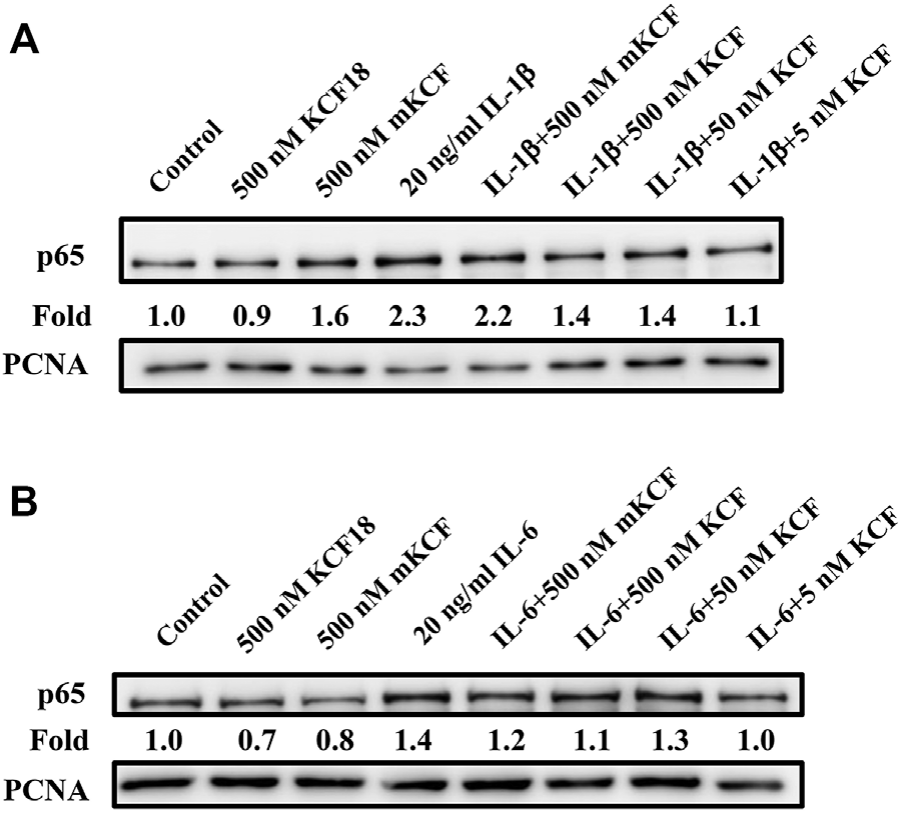
KCF18 alleviates the nucleation of p65 stimulated by cytokines in THP-1 cells. PMA-pretreated THP-1 cells were incubated with either IL-1β **(A)** and IL-6 **(B)** or by cytokines pretreated with KCF18 for 1 h. Four hours later, nuclear extracts were harvested, proteins were separated using SDS-PAGE, and P65 expression levels were determined using Western blotting.

**FIGURE 7:**
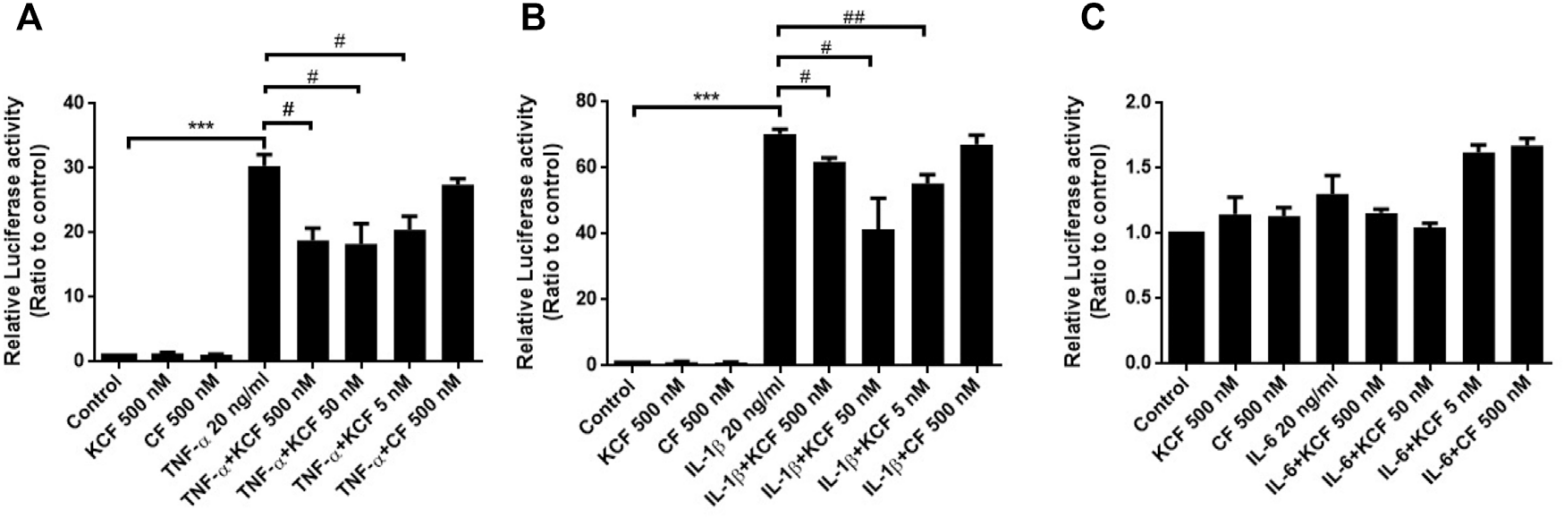
KCF18 suppresses the NF-κB luciferase activities stimulated by cytokines in HMEC-1 cells. The effects of KCF18 on **(A)** TNF-α-, **(B)** IL-1β-, and **(C)** IL-6-induced NF-κB luciferase activities were detected. Values are the mean ± SD of relative luciferase activities from three independent experiments performed in triplicate. ***p < 0.001 versus control, and #*p* < 0.05, ##*p* < 0.01, versus cells stimulated with cytokines in the presence of KCF18.

NF-κB activation plays a crucial role in cytokine-stimulated inflammation (Yamamoto and Gaynor, 2001). The main activated state of NF-κB is a heterodimer formed by p50 and p65, which usually combines with IκB to generate an inactive state in the cytoplasm. When the NF-κB pathway is activated, IκB will be phosphorylated and separated from NF-κB, allowing NF-κB to enter the nucleus and regulate target gene transcription. Therefore, we surveyed the participation of NF-κB in the inflammation-inhibitory effects of KCF18 in cells. To assess whether NF-κB activation by cytokines was suppressed through pretreatment with KCF18, Western blotting assay for p65, a subunit of NF-κB and a marker of its activation, was implemented. From the nuclear lysate of macrophages, IL-1β and IL-6 treatment induced p65 translocation into the nucleus 2.3- and 1.5-fold, respectively (Figures 6A,B). Pretreatment with 500, 50, and 5 nM KCF18 decreased the IL-1β-induced p65 translocation in a dose-dependent manner to 1.4-, 1.4-, and 1.1-fold, respectively. KCF18 pretreatment also reduced p65 nuclear translocation by 1.1-, 1.3-, and 1.0-fold for IL-6 stimulation (Figure 6B). However, the scramble peptide mKCF18 did not display the ability to inhibit p65 nuclear translocation. In addition, transient transfections were performed using an NF-κB-dependent luciferase reporter plasmid to further examine the effects of KCF18 on NF-κB transcriptional activity in endothelial cells. Treatment with TNF-α and IL-1β activated NF-κB luciferase in HMEC-1 cells (Figures 7A,B), whereas KCF18 effectively reduced TNF-α- and IL-1β-induced NF-κB luciferase activity. However, IL-6 did not induce NF-κB luciferase activity obviously (Figure 7C). These data indicate that KCF18 can regulate the translocation of p65 into the nucleus by binding to cytokines, thereby reducing the severity of the inflammatory response.

### Anti-Inflammatory Effect of KCF18 on the Expression of Proinflammatory Cytokine mRNA in the Liver of Mice With Endotoxemia

**FIGURE 8:**
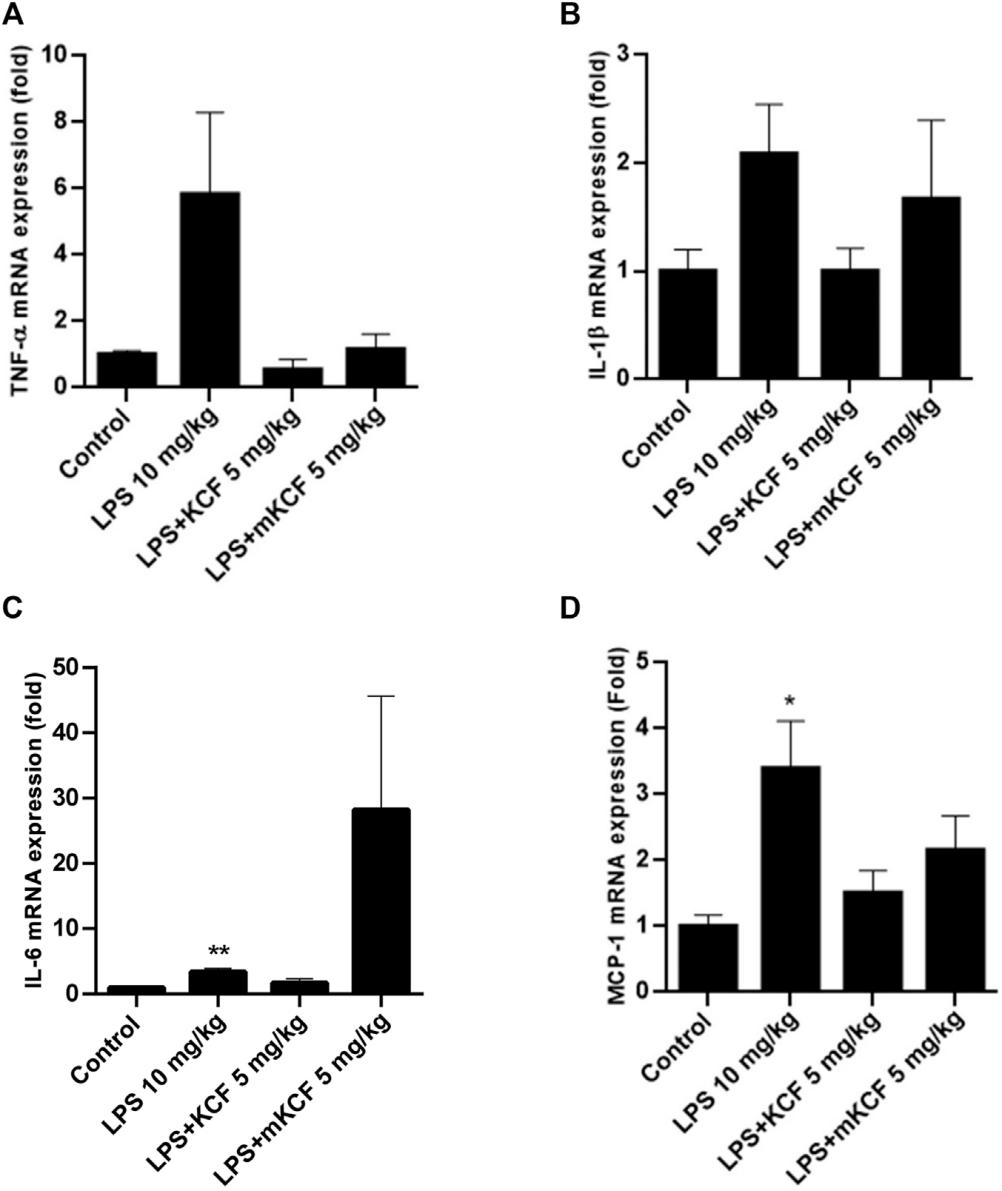
KCF18 reduced the expression of proinflammatory cytokine mRNA in the livers of mice with endotoxemia. In the LPS-induced endotoxemia mouse model, liver tissue was isolated, and TNF-α **(A)**, IL-1β **(B)**, IL-6 **(C)** and MCP-1 **(D)** mRNA levels were determined using qPCR assays as described in the Materials and Methods section. GAPDH cDNA was used as an internal control. Values are the mean ± SD of mRNA levels relative to those for GAPDH from three independent experiments. **p < 0.01, ***p < 0.001 versus control and #*p* < 0.05, ##*p* < 0.01 versus mice stimulated with LPS in the presence of KCF18 injection.

Sepsis is a systemic inflammatory disease caused by infection. Studies have indicated that the overexpression of cytokines is closely related to the severity of sepsis (Chaudhry et al., 2013). To evaluate the inflammation inhibitory activity of KCF18 in endotoxemia mice model, the TNF-α (Figure 8A), IL-1β (Figure 8B), IL-6 (Figure 8C), and MCP-1 (Figure 8D) mRNA levels in the mouse liver were determined using qPCR. TNF-α, IL-1β, IL-6, and MCP-1 mRNA levels in the liver sample were dramatically enhanced in the LPS-injected group compared with the normal group (*p* < 0.01; Figure 8). However, treatment with KCF18 at 5 mg/kg reduced LPS-induced IL-1β and IL-6 mRNA levels and significantly decreased TNF-α and MCP-1 mRNA levels when compared with the LPS group (*p* < 0.05). This indicated that KCF18 alleviated the mRNA expression levels of the inflammatory cytokines TNF-α, IL-1β, IL-6, and MCP-1 in an endotoxemia mouse model with liver injury.

### Anti-Inflammatory Effect of KCF18 on IL-6 Expression and White Blood Cell Count in the Blood of Mice With Endotoxemia

**FIGURE 9:**
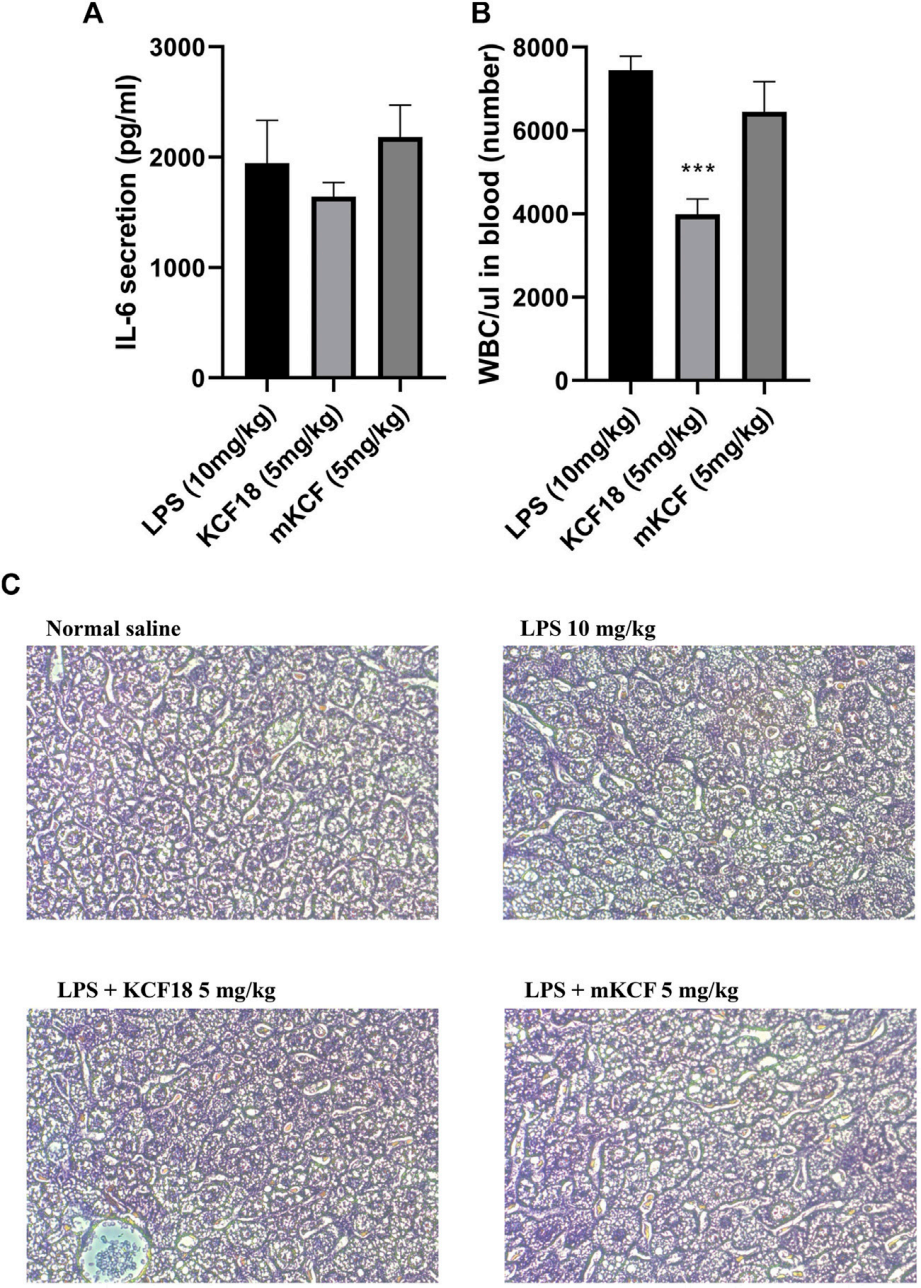
KCF18 reduced the IL-6 expression and WBC count in the blood of mice and alleviated tissue injury on liver histopathology with endotoxemia. LPS-induced endotoxemia mouse model was divided into Control, LPS, LPS + KCF18 (5 mg/kg) and LPS + mKCF (5 mg/kg) groups. IL-6 expression in the mice serum **(A)** was detected using an ELISA kit, WBC count **(B)** was determined, and histopathological changes in liver tissues **(C)** were observed under optical microscope by Hematoxylin and eosin staining, magnification 200×. LPS treatment mice had more severe liver injury and hepatocyte degeneration.

To further investigate the anti-inflammatory effects of KCF18 on LPS-induced endotoxemia in mice, the IL-6 expression level and white blood cell (WBC) count in the blood were detected. As shown in Figure 9A, IL-6 was induced in the LPS-injected group, and KCF18 reduced IL-6 expression in the KCF18-injected group. The WBC count was also decreased in the KCF18-injected group compared with the LPS-injected group (Figure 9B). However, mKCF did not express the inhibitory effects. This indicated that KCF18 could inhibit proinflammatory cytokine expression by reducing the expression of WBC in LPS-induced endotoxemia mice.

### Effect of KCF18 on the Tissue Injury of Liver Histopathology in Mice With Endotoxemia

To assess whether KCF18 could preserve liver tissue when such tissue is subjected to LPS-induced liver injury, a liver histopathological test was also performed. In the normal group, the liver tissues were intact, and the hepatocytes were regularly arranged, as depicted in Figure 9C. In the LPS group, the basic architecture of liver cells disappeared, and liver cells swelled. By contrast, treatment with KCF18 significantly improved the degree of liver histopathological alterations.

## Discussion

Sepsis is a disease caused by complex interactions between pathogenic bacteria and host immune response. Infection by pathogenic bacteria can cause the production of cytokine storms, which will destroy the integrity of the vascular endothelial layer, leading to hypotension and coagulation disorders and ultimately to tissue damage and organ failure (Chaudhry et al., 2013). Clinical studies have found that in patients with severe sepsis, the blood levels of TNF-α, IL-1β, IL-6, IL-8, IFN-γ, and MCP-1 are significantly increased (Bozza et al., 2007). An increase in the level of TNF-α in the serum of patients with sepsis increases the risk of mortality (Gogos et al., 2000); moreover, the level of IL-6 in the serum of patients with sepsis decreases, which helps patients with sepsis recover from the illness (Kumar et al., 2009). Therefore, reducing the cytokine levels in the plasma of septic patients is helpful for decreasing the severity and mortality of the patient and provides a suitable therapeutic method.

Many anti-inflammatory drugs that inhibit cytokines have been developed to retard the inflammation caused by pathogenic bacteria; however, the treatment of TNF-α neutralizing antibodies, soluble TNF receptors, and IL-1 receptor antagonists had no therapeutic effect on patients with sepsis in clinical trials (Clark et al., 1998; Dinarello, 2000). Therefore, it should not only inhibit one cytokine unilaterally. In addition, the purification and preparation of antibodies takes a long time and has a high production cost. Peptide drugs have a short production time and a high degree of specificity as well as predictable metabolic pathways. Therefore, peptide drugs may be the trend of future drug development. Previously, we used computer simulations to calculate the binding free energies of TNF-α, IL-1β, and IL-6 to their cognate receptors and designed a multifunctional peptide, KCF18, that can bind to TNF-α, IL-1β, and IL-6 (Jiang et al., 2019). We first explored whether KCF18 affects the expression of proinflammatory cytokines secreted by macrophages. As presented in Figure 2, when cytokines (TNF-α, IL-1β, and IL-6) are incubated with cells, they will increase the expression of TNF-α, IL-1β, and IL-6 mRNA and proteins in macrophages. However, pretreatment with various concentrations of KCF18 (500 nM, 50 nM, and 5 nM) can attenuate the mRNA and protein expressions of proinflammatory cytokines induced by TNF-α, IL-1β, and IL-6. Similar results were also observed with KCF18 pretreatment in endothelial cells by IL-1β and IL-6 stimulation (Figures 2E,F). However, the effect of KCF18 on TNF-α stimulation in endothelial cells is not as obvious as in macrophage (Figure 2D). The cause we cannot exclude may be the different cofactors or repressors which involve TNF-α receptor binding, express or bind on the surfaces of two cells to interfere the KCF18 interaction with TNF-α receptor in HMEC-1 cells. Since only 6 amino acids were designed for targeting TNF-α receptor binding, this interference by such cofactors or repressors might be enough to disrupt the binding of KCF18 to receptor. In contrast, the interference is not strong enough to interrupt the whole TNF-α molecule binding to its receptor. To discover whether the inhibitory effect of KCF18 on the decrease of proinflammatory cytokine expression induced by TNF-α, IL-1β, and IL-6 is due to the cytotoxicity to THP-1 cells, we used WST-1 reagent to confirm the cell growth status. As presented in Figure 3D, the cell viability of cocultivation of KCF18 with THP-1 cells did not extend the difference compared with the control group. This indicated that KCF18 decreased the transcription and expression of proinflammatory cytokines induced by TNF-α, IL-1β, and IL-6. However, in the experimental results, we observed that high concentrations of KCF18 were not necessarily the optimal inhibitory concentrations of cytokines. Sometimes a low KCF18 concentration had a significant inhibitory effect, meaning that the KCF18 effect may be unstable and may not be dose dependent or that the intensity of the message may not be directly proportional to the number of receptors (Bazzoni and Beutler, 1996). In addition, it is possible that a high KCF18 concentration promotes the formation of chemical bonds between peptides, causing the configuration of KCF18 to change, in which case the effect of KCF18 may not achieve the expected results. In addition, we also found that the most effective KCF18 concentration is not necessarily the same at the mRNA and protein levels. We speculated that this may be related to the posttranslational modification of cells and the protein degradation mechanism (Mann and Jensen, 2003; Vucic et al., 2011), which lead to different levels of mRNA and protein expression.

In the process of systemic inflammation, cytokines stimulate macrophages to release a large amount of ROS to remove foreign pathogens. When the concentration of ROS in tissue increases, the antioxidant capacity decreases or a large amount of reactive oxygen species is produced. This causes oxidative pressure in the tissue. In patients with severe sepsis and septic shock, a considerable amount of ROS is produced, which causes great oxidative pressure in tissues; in turn, this causes multiple organ failure (Crimi et al., 2006). In addition, active oxygen can interact with polyunsaturated fats. The acids interact with each other to increase the lipid peroxidation of fatty acids on the cell membrane, causing damage to the cell membrane function and affecting the normal physiological functions of the cell (Radi, 2013). Therefore, we explored whether KCF18 affects the expression of ROS secreted by macrophages under the stimulation of cytokines. The results shown in Figure 6 indicate that TNF-α, IL-1β, and IL-6 can promote the secretion of large amounts of ROS by macrophages, and when different concentrations of KCF18 are added, they could effectively inhibit the production of ROS induced by cytokines in a dose-dependent manner. In the study of Zughaier et al., positively charged antimicrobial peptides could reduce the TNF-α and NO secreted by human and mouse macrophages stimulated by LPS but could also promote the respiratory burst of macrophages (respiratory burst), which produces a large amount of ROS (Zughaier et al., 2005). However, our designed peptide can effectively reduce the production of ROS stimulated by cytokines. Therefore, compared with antibacterial peptides for treating sepsis, our designed peptide has greater therapeutic potential to reduce the severity of sepsis.

A large amount of ROS promotes the lipid peroxidation of LDL to form oxLDL. Studies have shown that oxLDL plays a key role in the damage of vascular endothelial function (Parthasarathy et al., 2010), and it also plays a vital role in the early development of arteriosclerosis (Li and Mehta, 2005; Parthasarathy et al., 2010). Therefore, reducing ROS production and LDL oxidation may help alleviate the development of early arteriosclerosis. To investigate whether KCF18 affects the ability of macrophages to engulf oxLDL, oxLDL uptake induced by cytokines was assessed. The addition of cytokines induced the ability of macrophages to phagocytose oxLDL (Figure 5). When cells were pretreated with KCF18, macrophage phagocytosis induced by cytokines was reduced. The results proved that KCF18 could retard the ability of macrophages to produce ROS and inhibit the phagocytosis of oxLDL. Therefore, in various cardiovascular diseases (such as atherosclerosis), the designed peptide may also have the potential for use in treatment.

NF-κB is an essential transcription factor that regulates inflammatory responses. When a pathogen invades, the LPS on the pathogen is recognized by the TLR4 receptor, triggering the transmission of downstream messages that activate the NF-κB pathway (Bachert and Geveart, 1999), which promotes cells to express a large number of cytokines—this amplifies the occurrence of inflammation. When NF-κB is activated, it facilitates the phosphorylation of p65 and enters the nucleus to regulate the DNA transcription of downstream genes. Studies have indicated that NF-κB in patients with sepsis increases greatly, which is related to a higher mortality rate and slower recovery after illness (Bohrer et al., 1997; Arnalich et al., 2000; Liu and Malik, 2006). Therefore, we explored whether the reduction of cytokine production by KCF18 was affected by inactivation of the NF-κB pathway. Figure 7 shows that when cytokines stimulated the cells, the nuclear translocation of p65 was increased. However, the induction of p65 translocation was suppressed when KCF18 was pretreated with cells. A similar result was also observed in endothelial cells when the NF-κB-dependent luciferase reporter assay was conducted.

A lower KCF18 concentration exhibited a superior inhibitory ability. Surface charge may affect macrophage phagocytosis. The negatively charged sialic acid on the surface of macrophages can bind to positively charged nanoparticles and phagocytose the nanoparticles into the cell (Gallagher et al., 1987). Charged nanoparticles are also more likely to be swallowed by macrophages than are uncharged nanoparticles (Roser et al., 1998; Xiao et al., 2011). Therefore, a higher concentration of positively charged KCF18 (Figure 1) may accelerate macrophage phagocytosis, thereby reducing the effect of KCF18. This needs to be clarified in the future.

The biggest disadvantage of peptide drugs is their short half-life in the blood and low bioavailability. However, in the mice endotoxemia model, as expected, LPS injection indeed upregulated the expressions of TNF-α, IL-1β, IL-6, and MCP-1 mRNA. In contrast, KCF18 decreased LPS-induced expressions of TNF-α, IL-1β, IL-6, and MCP-1 mRNA in the liver even though no statistical difference (Figure 8). The reason may be due to the individual differences in mice. Another evidence was also proofed that WBC count in the blood was reduced by KCF18 (Figure 9B). The liver tissue in the normal control group displayed normal hepatocytic architecture. Conversely, a remarkable liver pathological changes such as vacuolization, and liver lobule destruction were observed in LPS group. The liver lobules of the mice in the LPS/w KCF18 group had less liver tissue structural damage, with clearer liver lobules (Figure 9C). The results confirmed that KCF18 decreased the severity of inflammation in the endotoxemia model. Gutsmann et al. designed anti-lipopolysaccharide peptides and revealed available suppression of LPS-induced cytokine secretion and conservation from lethal septic shock *in vivo* (Gutsmann et al., 2010). On the other hand, our collaborator Shih et al. also showed that KCF18 can alleviate liver injury in endotoxemia mice (Shih et al., 2021). In another study they used a peptide that only targets TNF-α and eased liver injury to mice (Chang et al., 2020). It indicates that peptide drugs have excellent potential for future pharmacological application. Our designed peptide KCF18 was derived from the complex structures of cytokine-receptor, which could target three major proinflammatory cytokines, TNF-α, ΙL-1β, and IL-6. Due to the complexity of inflammation responses and specificity of ligand-protein binding, peptide KCF18 could not target all inflammation-related cytokines, but only these three major proinflammatory cytokines. In addition, the limitation of peptide drugs is their short half-life in the blood; therefore, a new conjugation material might be constructed to ameliorate this problem. In the previous reports, Baig’s group showed that signaling pathways through toll-like receptor 4 (TLR4) stimulate several transcription factors such as NF-κB, Signal Transducers and Activators of Transcription family of transcription factors (STAT1), Interferon regulatory factors (IRF’s), and activator protein 1 (AP1), which are the major mediators regulating the inflammatory response (Roy et al., 2016). They claimed that repression of these objectives and their upstream signaling molecules supplies a latent therapeutic approach to cure inflammatory diseases. Meng’s group also showed that endotoxin lipopolysaccharides via TLR4 signaling pathway to activate translocation of NF-κB and further regulate expression of pro-inflammatory genes, including TNF-α, IL-6, and IL-1β during sepsis (Jiang et al., 2015). Similarly, KCF18 could alleviate inflammation mediated by blocking the interactions of TNF-α, IL-6, and IL-1β with their cognate receptors, reducing the translocation of NF-κB and reduction the inflammatory gene expressions. This proved that KCF18 can prevent inflammation and may provide a new direction and therapeutic treatment method for inflammation-related vascular diseases such as sepsis.

## Data Availability

The original contributions presented in the study are included in the article/Supplementary Material, further inquiries can be directed to the corresponding authors.
