# Supplementary material for: A Multitarget Therapeutic Peptide Derived From Cytokine Receptors Based on in Silico Analysis Alleviates Cytokine-Stimulated Inflammation
Source: Front Pharmacol. 2022 Mar 10;13:853818. doi: 10.3389/fphar.2022.853818 (PMC8965626; doi:10.3389/fphar.2022.853818)
Supplement: Supplementary file 1 [file DataSheet1.PDF]

Supplementary information

A multitarget therapeutic peptide derived from cytokine receptors based on in silico analysis alleviates cytokine-stimulated inflammation

Chun-Chun Chang[1,2], Shih-Yi Peng[3], Hao-Hsiang Tsao[3], Hsin-Ting Huang[3], Xing-Yan Lai[4], Hao-Jen Hsu[4,*], Shinn-Jong Jiang[3,*]

[1]Department of Laboratory Medicine, Hualien Tzu Chi Hospital, Hualien, 97004 Taiwan
[2]Department of Laboratory Medicine and Biotechnology, College of Medicine, Tzu Chi University, Hualien, 97004 Taiwan
[3]Department of Biochemistry, School of Medicine, Tzu Chi University, Hualien, 97004 Taiwan
[4]Department of Life Sciences, College of Medicine, Tzu Chi University, Hualien, 97004 Taiwan

*** Correspondence:**
Hao-Jen Hsu, Email: hjhsu32@mail.tcu.edu.tw

Shinn-Jong Jiang, Email: sjjiang@mail.tcu.edu.tw

Co-corresponding authors.

#Contributed equally.

Fig. S1

A

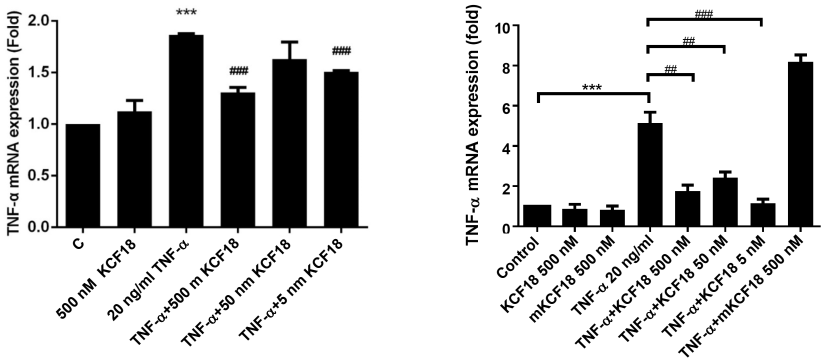

Figure S1. KCF18 downregulates cytokines-induced mRNA transcripts of TNF-α in cells. PMA-pretreated THP-1 cells (A) or HMEC-1 cells (B) were cultivated with either cytokines at a concentration of 20 ng/ml, or by cytokines pretreated with KCF18 for one hour. 4 hours later, total RNA was isolated, and analysis of cytokines mRNA levels were determined using qPCR assays. GAPDH cDNA was used as an internal control. Values are the mean ± S.D. of mRNA levels relative to those for GAPDH from three independent experiments. *P < 0.05, ** P <0.01, *** P <0.001 vs. control and # P < 0.05, ## P <0.01, ### P <0.001 vs. cells stimulated with cytokines in the present of KCF18.
